# Beneficial Effects of Oleosomes Fused with Human Fibroblast Growth Factor 1 on Wound Healing via the Promotion of Angiogenesis

**DOI:** 10.3390/ijms232113152

**Published:** 2022-10-29

**Authors:** Yongxin Guo, Guodong Chu, Weijia Cai, Yaying Li, Xinxin Lan, Jing Li, Linna Du, Jing Yang

**Affiliations:** Engineering Research Center of Bioreactor and Pharmaceutical Development, Ministry of Education, College of Life Science, Jilin Agricultural University, No. 2888, Xincheng Street, Changchun 130118, China

**Keywords:** oleosomes, wound, angiogenesis, human umbilical vein endothelial cells, proliferation

## Abstract

In our previous study, human fibroblast growth factor 1 was successfully fused with oleosomes, energy-storing organelles of seeds, which are considered to be excellent “expression carriers” for substances with a convenient purification process. The present work aimed to explore the beneficial effects of oleosomes fused with human fibroblast growth factor 1 (OLAF) on wound healing. The data showed marked improvements in terms of the angiogenesis, vascular integrity, collagen and inflammation on the wound sites of rats with a full-thickness skin defect. Moreover, the positive role of OLAF in promoting angiogenesis and its possible pathways were clarified in vivo and in vitro. The results showed that the number, length and branches of the blood vessels of the chick embryo chorioallantoic membrane were markedly increased after OLAF treatment. Meanwhile, the in vitro results also revealed that 100 ng/mL OLAF exhibited a promoting effect on the proliferation, migration and tube formation of human umbilical vein endothelial cells. In addition, the potential of OLAF to improve wound angiogenesis was demonstrated to be associated with an up-regulated PI3K/Akt pathway by transcriptome sequencing analysis and the introduction of a PI3K/Akt pathway inhibitor (LY294002). These findings suggest that OLAF has many prospects in the development of drugs for wound healing.

## 1. Introduction

Healthy skin with structural integrity is an important necessity for maintaining the physiological stability of the body [1]. However, skin injuries, which can be caused by numerous incidences, such as trauma, surgical operations and chronic diseases, have become a major medical problem worldwide [2]. Injured skin can be regenerated through a complex, biologically dynamic self-healing process [3]. Nonetheless, many factors can affect the remodeling of injured skin, resulting in the slow and poor healing of wounds [4]. Delayed wound healing further aggravates the risk of bacterial infection and can even cause death. Meanwhile, it also inflicts heavy physiological and psychological burdens on patients [5]. Therefore, the transdermal delivery of drugs to accelerate the reconstruction of injured skin is highly desirable in clinical applications.

In recent years, a decrease in blood nourishment has been considered to be the main reason for the increased risk of infection and delayed healing of wounds [6]. A plentiful vascular supply in the skin can provide a sufficient amount of oxygen, nutrients and active factors for the tissue and remove metabolic waste, which means that the remodeling of the wound’s vascular network becomes the fundamental requirement for tissue to regenerate properly [7,8,9]. Normally, the body can maintain the dynamic balance between the formulation and inhibition of blood vessels [10]. During angiogenesis, the vasculature begins to actively proliferate, which has proved to be an important factor affecting the efficiency of wound repair [11]. Angiogenesis is interpreted as the development of a new stable vascular network, formed by new vessels sprouting from the parent blood vessels [12,13]. This process seems to be a dynamic multistep process regulated by pro- and anti-angiogenic factors, including the activation, migration and proliferation of endothelial cells; lysis of the basement membrane; elongation and anastomosis of sprouting vessels; restoration of the basement membrane; and maturation of functional capillaries. Impaired angiogenesis seriously affects the remodeling of the vascular network [14,15,16,17]. Thus, searching for new drugs that promote angiogenesis is crucial for the treatment of wounds.

Acidic fibroblast growth factor (FGF1), a well-known member of the FGF protein family, has been shown to play a strong role in neuronal regeneration, the relief of diabetic hyperglycemia, etc. [18]. However, its short half-life and instability at room temperature severely limit the use of FGF1, to a certain extent [19,20]. With the commercial production of several plant-derived pharmaceutical proteins, extensive attention has been focused on the application of the plants as foreign substance production factories [21]. Through the previous pioneering work of our group, multiple recombinant proteins were successfully expressed using seed-based platforms [22,23]. Increasing evidence shows that the physiological quiescence state and low water content of mature seeds enable fusion proteins to be stably stored in seeds for a longer time [24]. In our previous studies, oleosin fusion technology was selected to express foreign proteins, in which the proteins were fused with the N and/or C termini of the oleosins and accumulated in the energy-storing organelles found in seeds, namely, oleosomes (i.e., oil globules) or oil bodies [25]. Oleosomes lend themselves well to biotechnological applications in agriculture, medicine and industry owing to their useful properties, i.e., their simplified protein isolation, long-term preservation in seeds and good transdermal absorption capacity [26,27,28]. In addition, oleosomes are abundant in redox-active prenyl-lipids, which have been proved to have an excellent scavenging effect on reactive oxygen species [29]. These characteristics render oleosomes as good carriers of transdermal proteins. In our recent works, oleosomes that accumulate the human fibroblast growth factor 1 (hFGF1) were also obtained using this platform, and it was proved that they have a good safety after transdermal administration [30,31]. Moreover, the transportation mode and storage conditions of these recombinant seeds are very convenient. However, the activity of these recombinant oleosomes in accelerating wound healing and its mechanism are still unknown.

Herein, the wound healing potential of oleosomes fused with hFGF1 (OLAF) in rats with a full-thickness skin defect was first investigated. On this basis, the promoting effect of OLAF on the angiogenesis of wounds and its mechanism were further studied.

## 2. Results

### 2.1. The Wound Healing Potential of Oleosomes Fused with Human Fibroblast Growth Factor 1 in Rats

**Figure 1:**
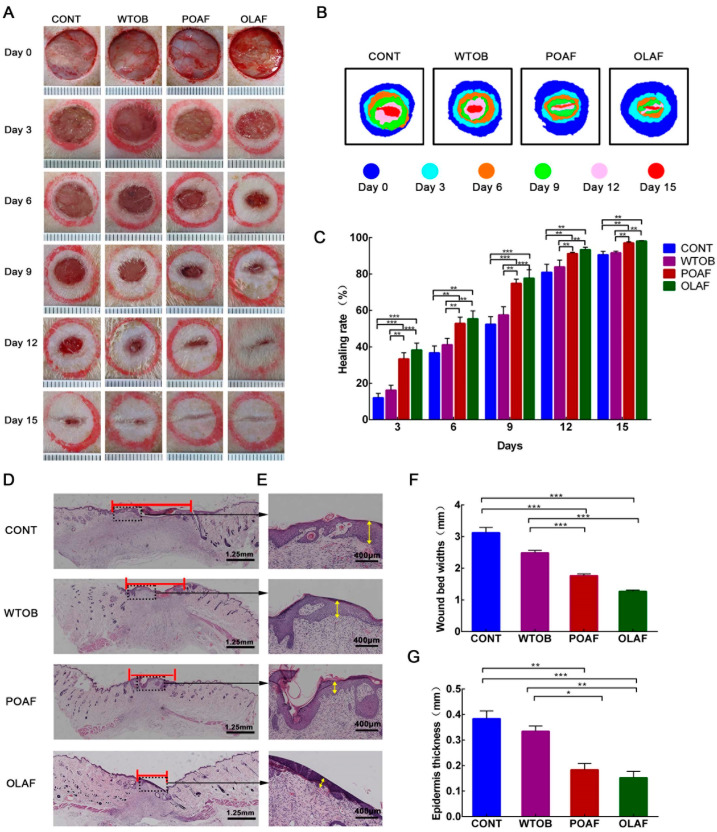
Transdermal treatment with oleosomes fused with human fibroblast growth factor 1 (OLAF) promoted wound repairing in rats with a full-thickness skin defect. (**A**) Macroscopic view of wound sites treated with test substances over 15 days. (**B**) Heat maps were introduced to simulate the changes in the wound closure. (**C**) Quantitative analysis of the wound healing rates of the different groups. (**D**) Histological diagrams of wound sections stained with H&E on day 15 and their enlarged images, where the margin of the wound is indicated by the red line. (**E**) Higher magnification of black dotted squares for observing the epidermal thickness, which is indicated by the yellow arrow. (**F**,**G**) Quantification of the wound bed widths and epidermal thickness. * *p* < 0.05, ** *p* < 0.01, *** *p* < 0.001. All obtained data are displayed in the form of mean ± SD (n = 6).

No animal deaths or adverse reactions were observed over 15 days. On days 0, 3, 6, 9, 12 and 15, the wounds of the experimental rats were photographed, and the quantitative analysis of the wound healing rate was performed. With the prolongation of the treatment time, it was observed that the wound areas gradually decreased in all of the groups (Figure 1A). This was particularly evident in the OLAF and POAF groups. On the 15th day after the operation, the OLAF- and POAF-treated wounds were almost closed, whereas the wounds in the other groups showed obvious unhealed areas. To simulate the wounds of the rats, heat maps were generated, the results of which were consistent with the gross appearance of wounds (Figure 1B). The abovementioned results obtained from the morphological observations suggest that the wounds that received OLAF exhibited improved wound closure. Meanwhile, compared with the CONT group, at each time point, the healing rate of the wounds of the OLAF-treated rats significantly increased (*p* < 0.01), which was also markedly higher than that of the WTOB group (Figure 1C, *p* < 0.01). After 15 days of the OLAF treatment, the wound healing rate of the rats had already reached 98.06%. It should be noted that the WTOB group showed a higher wound healing rate than the CONT group, but the difference was not obvious (Figure 1C, *p* > 0.05).

To further analyze the pathological changes in the skin tissue, H&E staining of the wound sections was introduced. As evidenced by the H&E staining, the wounds that received OLAF appeared to be smaller compared to the physiological-saline-treated wounds at day 15 after the operation, which was further proved by the quantification analysis of the wound bed widths (Figure 1D,F). Meanwhile, relative to the CONT group, the thicknesses of the epidermis in the POAF- and OLAF-treated groups were markedly reduced by approximately 48% and 51%, respectively (Figure 1E,G). All of the above results reveal that OLAF are capable of promoting wound healing.

### 2.2. The Effects of Oleosomes Fused with Human Fibroblast Growth Factor 1 on the Collagen Deposition and CD68+ Macrophage Infiltration of Wounds

**Figure 2:**
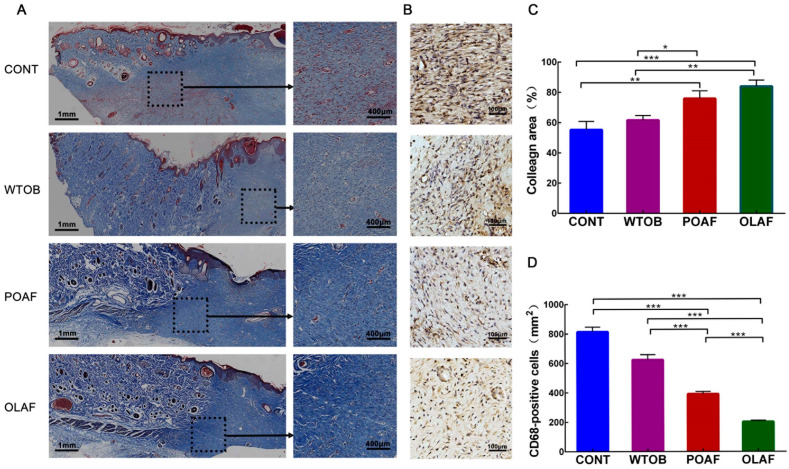
Effect of oleosomes fused with human fibroblast growth factor 1 (OLAF) on collagen deposition and macrophages at the wound sites. (**A**) Images of stained collagen fibers in wound sites harvested at 15 days post-wounding (blue color), where the collagen areas adjacent to the wound edge are indicated by the black dotted line. (**B**) Immunohistochemical staining showing CD68 expression in the wounds after 3 days treatment (brown color). Scare bar: 100 μm. (**C**) Quantification of the collagen content per microscopic field in wound tissue collected from different groups at 15 days post-exposure. (**D**) Density analysis of CD68-positive cells (per mm^2^). * *p* < 0.05, ** *p* < 0.01, *** *p* < 0.001. The mean ± SD was employed to display all the obtained data (n = 6).

As the main component of connective tissue, collagen synthesis can improve the strength of tissues, which is considered a key event during wound healing [32]. In this study, the collagen deposition at the wound sites in each group was determined by Masson’s trichrome staining. In the case of the OLAF- and POAF-treated groups, an increased staining intensity of the collagen fibers was observed at the site of the wound enclosure (Figure 2A). Meanwhile, the quantification analysis revealed that the collagen area in the OLAF group was evidently higher than that in the CONT and WTOB groups (CONT: *p* < 0.001 and WTOB: *p* < 0.01 vs. OLAF, respectively, Figure 2C). These results suggest that the collagen deposition in the wounds accelerated after 15 days of OLAF treatment.

Due to the function of the macrophages in the modulation of the local inflammatory microenvironment and in the resolution of inflammation, they are particularly important for the repair of wounds [33]. Herein, immunohistochemistry technology was used for the detection of the expression of the macrophage marker (CD68+). With the extension of treatment, the ratio of CD68-positive cells in the wounds treated with different substances decreased gradually (Figure S1). Through the analysis of the CD68-positive cells in the wounds at day 3, it seemed that more CD68-positive cells were found in the wounds exposed to physiological saline. In contrast, the number of CD68-positive cells in the OLAF and POAF groups obviously decreased (OLAF: *p* < 0.001 and POAF: *p* < 0.001 vs. CONT, respectively, Figure 2B,D). It is worth mentioning that a notable difference was observed in the CD68-positive cells between the OLAF group and the POAF group (*p* < 0.001). These data reveal that OLAF might be involved in the regulation of inflammation in wounds.

### 2.3. Oleosomes Fused with Human Fibroblast Growth Factor 1 Facilitated Angiogenesis at the Wound Sites

To analyze the effects of OLAF on angiogenesis during wound repair, immunohistochemistry staining for CD31 (a marker of endothelial cells) was conducted. As demonstrated in Figure S2, the peak in the CD31 expression appeared on the 9th day of wound healing, and at this time point, the OLAF group showed a higher number of CD31+ cells in relation to the control group.

**Figure 3:**
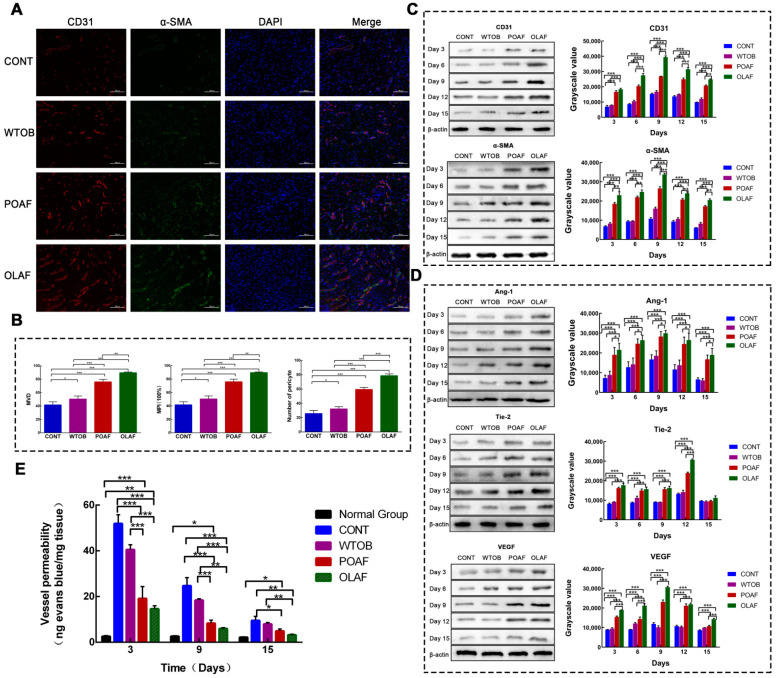
Evaluation of the effect of oleosomes fused with human fibroblast growth factor 1 (OLAF) on angiogenesis in rats with a full-thickness skin defect. (**A**) Representative immunofluorescence of wounds that received different substances for 9 days post-wounding for detecting CD31 (red), α-SMA (green) and DAPI (blue). Scare bar: 100 μm. (**B**) Statistical analyses of micro-vessel density (MVD), number of pericytes and the microvascular pericyte coverage index (MPI). (**C**,**D**) Western blot and quantitative analyses of CD31, α-SMA and pro-angiogenic factors at different times post-injury. (**E**) Effect of OLAF on the vascular permeability in healed wounds. * *p* < 0.05, ** *p* < 0.01, *** *p* < 0.001. The mean ± SD was employed to display all the obtained data (n = 6).

Subsequently, double immunofluorescence staining for CD31 and alpha smooth muscle actin (α-SMA, a marker of pericyte) was further performed to detect the angiogenesis on the 9th day of treatment. As demonstrated in Figure 3A, a small number of individual CD31+ cells appeared in the newly formed granulation tissue of rats in the CONT group, some of which were surrounded by α-SMA+ pericytes. In contrast, a larger number of CD31+ and α-SMA+ cells were found in the OLAF- and POAF-treated wounds, with the highest increase observed after the OLAF treatment. Furthermore, quantitative analyses of the number of α-SMA+ pericytes, MVD counts and MPI were conducted. It can be seen that the pericyte counts, MVD and MPI in the OLAF group were markedly higher than those in the CONT group by 4.1, 2.1 and 2 times, respectively (*p* < 0.001, Figure 3B). Moreover, the expressions of CD31, α-SMA and pro-angiogenic factors (i.e., VEGF, Ang-1 and Tie-2) were both significantly upregulated in the OLAF-treated wounds according to the Western blot analysis (Figure 3C,D). Taken together, these findings reveal that the beneficial effects of OLAF on wound repair might be related to increased angiogenesis.

### 2.4. The Effect of Oleosomes Fused with Human Fibroblast Growth Factor 1 on Vascular Permeability in Rats

To assess whether OLAF also protected the integrity of the newly formed vascular networks in the wounds, the extravasated Evan’s blue dye applied from the circulation to wound granulation tissue was measured (Figure 3E). On the 3rd, 9th and 15th days of treatment, the Evan’s blue diffusion was reduced substantially in the OLAF-treated wounds compared to the wounds that received physiological saline treatment (*p* < 0.01). After 9 and 15 days of administration, no obvious differences were found in the concentrations of Evan’s blue in the OLAF-treated tissue compared with the normal group (*p* > 0.05). This reveals that OLAF are capable of reducing vascular leakage and improving vascular integrity in the wound sites.

### 2.5. The Oleosomes Fused with Human Fibroblast Growth Factor 1 Stimulated Angiogenesis on CAM

**Figure 4:**
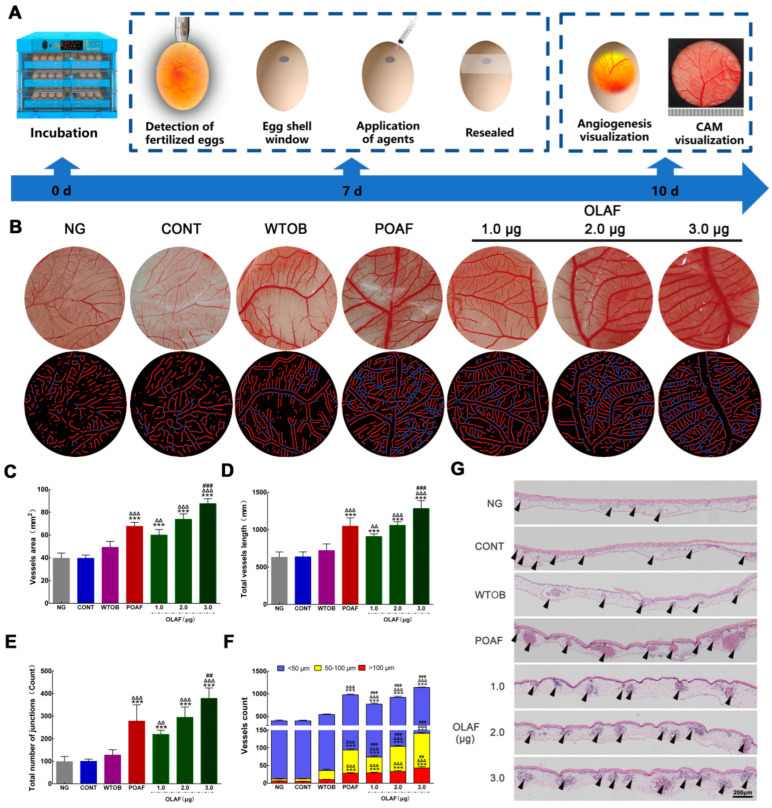
Oleosomes fused with human fibroblast growth factor 1 (OLAF) accelerated angiogenesis in the chick embryo choriollantoic membrane (CAM). (**A**) A schematic illustration of the chick embryo choriollantoic membrane assay used to investigate the angiogenesis induced by OLAF. (**B**) Appearance observation (first row) and simulation diagram of the blood vessels (second row) of the chicken embryo allantoic membrane in seven groups, where the red lines and blue dots are used to indicate the blood vessels and junctions, respectively. (**C**–**F**) Statistical charts of the vessel area, total vessel length and the number of vessel junctions and blood vessels with different diameters in the chorioallantoic membrane of the chicken embryo. (**G**) Histological morphology of the chicken chorioallantoic membrane was observed to assess the differences in the blood vessels between various groups, which are marked by black arrows. The mean ± SD was employed to display all the obtained data (n = 6). *** *p* < 0.001 vs. CONT group; ^ΔΔ^ *p* < 0.01, ^ΔΔΔ^ *p* < 0.001 vs. WTOB group; ^##^ *p* < 0. 01, ^###^ *p* < 0.001 vs. POAF group.

The pro-angiogenic potential of OLAF in the CAM was further studied by analyzing the morphological and histological changes in the CAM following the samples’ treatment, as shown in Figure 4A. The results regarding the blood vessels’ appearance and the simulation diagram showed obvious increases in the number of blood vessels (red lines) and junctions (blue points), which were observed in the CAM exposed to higher concentrations of OLAF (Figure 4B). The quantification of the macro-image analysis of the CAM showed that the POAF and 3.0 μg OLAF treatments increased the vessel area by 71% and 121%, respectively, compared with the CONT group (Figure 4C). Moreover, the POAF and 3 μg OLAF treatments caused a 1.66- and 2.04-times higher total vessel length than the CONT group (Figure 4D). Similarly, higher total numbers of junctions were found in the OLAF group and POAF group (Figure 4E). Furthermore, the numbers of blood vessels with different diameters were analyzed quantitatively. The POAF and OLAF treatments significantly increased the number of blood vessels with different diameters (i.e., <50, 50–100 and >100 μm) to varying degrees (Figure 4F, *p* < 0.001). H&E staining of the CAM sections was conducted, and the results reveal that OLAF markedly increased the vascular plexus compared to the control (Figure 4G). Taken together, the obtained results confirm the pro-angiogenic potential of OLAF.

### 2.6. OLAF-Induced Cell Proliferation, Scratch Wound Healing, Migration and Tube Formation in HUVECs

**Figure 5:**
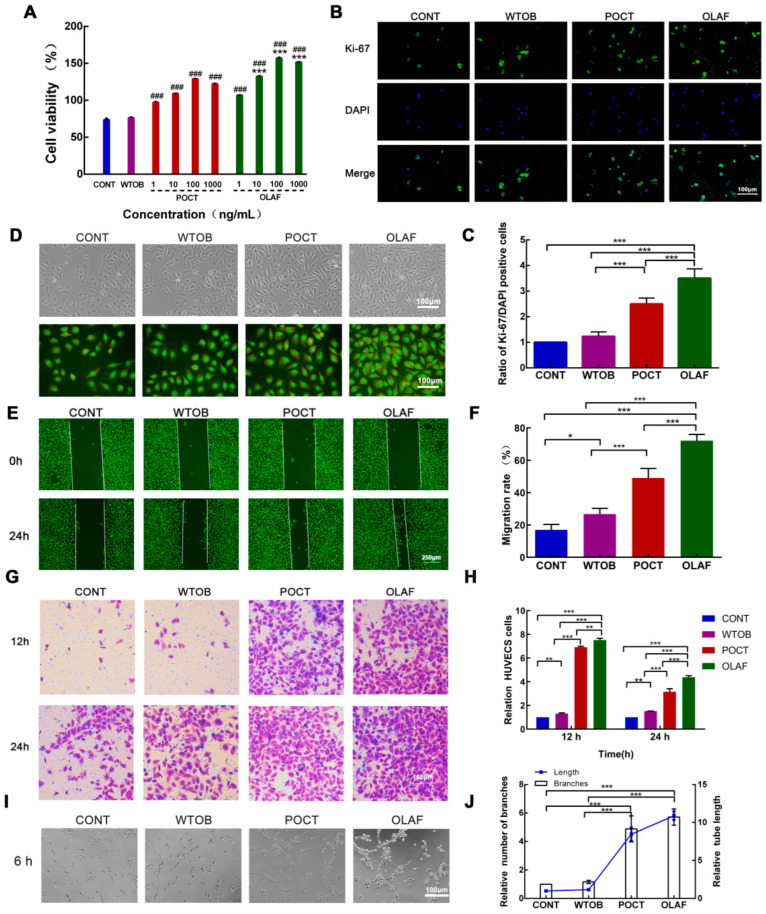
Effect of oleosomes fused with human fibroblast growth factor 1 (OLAF) on angiogenesis in vitro. (**A**) Cell viability assay was introduced to detect the viability of OLAF-exposed HUVECs. ^###^ *p* < 0.001 vs. CONT group; *** *p* < 0.001 vs. the samples of POCT group with the same concentration. (**B**) Images of immunofluorescence staining of Ki-67-positive HUVECs in various groups. (**C**) Bar chart demonstrating the ratio of Ki-67-positive HUVECs. (**D**) Representative morphological images of unstained (first row) and acridine-orange-stained (second row) HUVEC cells in different groups. (**E**) A scratch test was executed to investigate the migration of cells exposed to OLAF. (**F**) Quantitative analysis of the cell migration rate. (**G**) A cell invasion assay was carried out to verify the invasion of the HUVECs after OLAF treatment. (**H**) Quantification results of the migration test. (**I**) The effect of OLAF on the tube formation of the HUVECs. (**J**) A statistical chart of the tube length and relative number of branches in the tube formation assay. * *p* < 0.05, ** *p* < 0.01, *** *p* < 0.001. Mean ± SD was employed to display all the obtained data (n = 6).

To determine the pro-angiogenic activity of OLAF, different in vitro assays were used. Since the proliferation of endothelial cells is essential for effective wound healing, as mentioned above, the proliferation of the HUVECs was first detected using the CCK-8 assay [34]. As portrayed in Figure 5A, the viability of the OLAF- and POCT-exposed HUVECs was higher in relation to the CONT group and WTOB group. Twenty-four hours of incubation with 100 ng/mL OLAF produced the highest increase in the HUVEC cell viability, which increased by approximately 156.9% versus the CONT group. Moreover, immunofluorescence staining of the Ki-67 protein was employed to verify the promoting effects of 100 ng/mL OLAF on the HUVECs’ proliferation. It was found that the OLAF group presented the highest number of Ki67-positive cells, which was markedly higher than that of the other groups (*p* < 0.001, Figure 5B,C). At the same time, the morphology of the cells in the different groups was observed using a fluorescence inverted microscope. No obvious differences in the cell morphology were found between treatment groups, while the cells in the OLAF group were observed to be denser (Figure 5D). These results indicate that OLAF exhibited a promotion of the proliferation of the HUVECs.

Since the migration ability of vascular endothelial cells is another key factor affecting vascular bud growth and lumen formation in angiogenesis, scratch tests and transwell migration assays were performed. After the incubation of the HUVECs with individual OLAF or POCT for 24 h, the migration rate was measured. The statistical analysis revealed that the OLAF-treated cells exhibited a migration rate of 72.2% in comparison with the migration rates of 26.75% and 49.08% observed in the WTOB group and POCT group, respectively. In contrast, a migration rate of 16.84% was observed in the untreated HUVECs (Figure 5E,F). Moreover, the transwell detection tests showed that after treatment for 24 h, the relative number of migrated HUVECs in the OLAF group was 4.80, 3.15 and 1.50 times higher than that in the CONT, WTOB and POCT groups, respectively (Figure 5G,H). Collectively, these findings demonstrate the promoting effect of OLAF on the migration of HUVECs.

**Figure 6:**
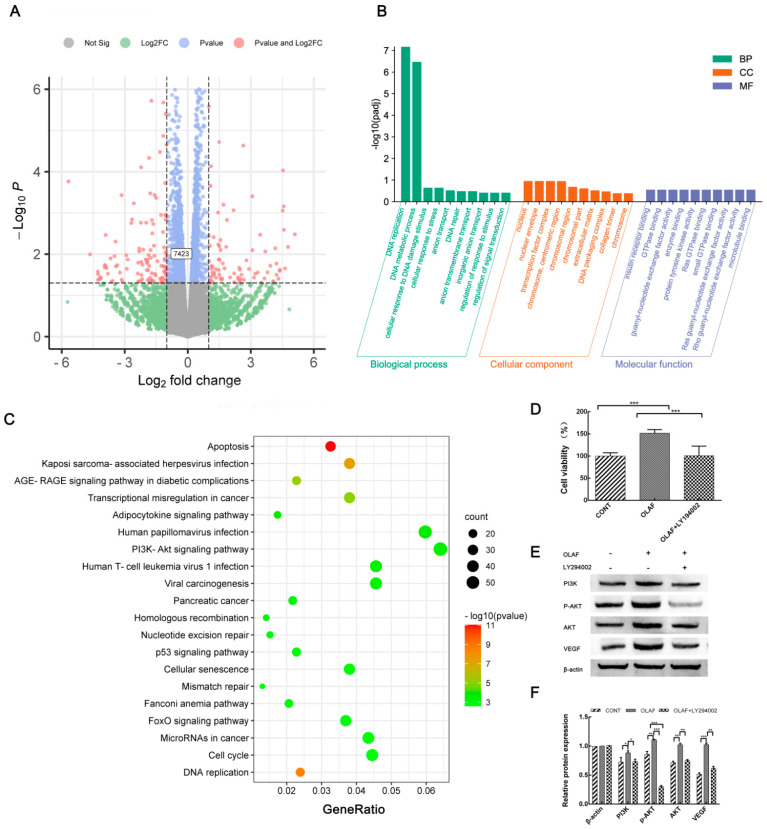
Effects of oleosomes fused with human fibroblast growth factor 1 (OLAF) on the PI3K/AKT pathway in the HUVECs and transcriptome analysis. (**A**) Volcano plot of differentially expressed genes (DEGs) between OLAF-treated and untreated HUVECs. Grey points represent the genes with no significant difference between the two groups. Green points and purple points represent genes with significant differences in the Log2Fold charge or *p* value between groups, respectively, and orange points are used to indicate the genes with significant differences in the *p*-value and Log2Fold charge between groups. The unigene 7423 is annotated as VEGF. (**B**) The top 10 GO terms for the biological process (BP), cellular component (CC) and molecular function (MF) in the GO enrichment analysis of the DEGs. (**C**) The top 20 KEGG pathways enriched by DEGs. (**D**) LY294002 changed the promoting effect of OLAF on the viability of HUVECs. (**E**,**F**) Changes in the expression of PI3K-AKT-pathway-related proteins in HUVECs stimulated by OLAF with or without LY294002, and the corresponding statistical analysis results. * *p* < 0.05, ** *p* < 0.01, *** *p* < 0.001. Mean ± SD was employed to display all the obtained data (n = 3).

Additionally, to elucidate the tube- and capillary-forming ability of the HUVECs treated with the samples, a capillary-like tube formation assay was used. It can be seen that the addition of OLAF or POCT stimulated the HUVECs’ tube formation (Figure 5I). The morphometric measurements revealed that the treatment of the HUVECs with OLAF promoted tube formation, with a maximal 5.7- and 10.9-fold increase in the relative number of branches and relative tube length, respectively, compared with the vehicle control (Figure 6J). These data prove that OLAF accelerated tube formation in the HUVECs.

Moreover, qRT-PCR was performed to investigate the relative expression levels of three genes (*VEGF*, *Tie*, *Ang-1*) of the cells, and obvious higher expressions of *VEGF*, *Tie* and *Ang-1* were found in the OLAF-treated cells (Figure S3).

### 2.7. OLAF Improved Angiogenesis through the PI3K-AKT Pathway

To analyze the gene expression profiles of the proteins in different angiogenesis-related pathways, the transcriptome analyses of the HUVECs treated and untreated with OLAF were carried out. Overall, 2137 DEGs were identified, 1070 of which were upregulated and 1067 of which were downregulated following OLAF treatment compared to the control cells (Figure 6A). The DEGs were further utilized for the GO term analysis, the results of which showed that 716, 386 and 1150 DEGs were enriched in the biological processes, cellular components and molecular functions, respectively. The top 10 enriched GO terms within each subcategory are shown in Figure 6B. Additionally, 921 DEGs were enriched in the 310 KEGG pathways, including the FoxO signaling pathway, PI3K/Akt signaling pathway and p53 signaling pathway, and the top 20 enriched KEGG pathways of the DEGs are shown in Figure 6C. Among these 20 pathways, the number of DEGs (59) enriched in the PI3K/Akt pathway was the largest, implying that the function of OLAF in accelerating angiogenesis might be related to the PI3K/Akt signaling pathway. To explore the role of the PI3K/Akt signaling pathway in angiogenesis induced by OLAF, LY294002 (a specific PI3K inhibitor) was introduced to pretreat the HUVECs before OLAF stimulation. As shown in Figure 6D, the pretreatment with 10 μmol/L LY294002 attenuated the promotive effect of OLAF on the HUVECs (*p* < 0.001). These results elucidated that the PI3K/Akt pathway might be involved in the modification of the cellular proliferation behavior of HUVECs cells. Moreover, the Western blot detection revealed that OLAF markedly promoted the expression of VEGF, as well as the activation of the PI3K/Akt pathway, by detecting the expression of PI3K and Akt and the phosphorylation of Akt (p-Akt) in the HUVECs treated with OLAF, which could be inhibited by pretreatment with LY294002 (Figure 6E,F). Taken together, the above data suggest that the pro-angiogenic activity of OLAF might be explained by the upregulation of the expression of VEGF through the activation of the PI3K/Akt signaling axis.

## 3. Discussion

In the present study, the wound-healing-promoting potential of oleosomes fused with fibroblast growth factor 1 (OLAF), a new transdermal drug obtained in our previous study, was investigated in full-thickness skin defect rats. Their positive effects on wound closure, inflammatory, collagen deposition and angiogenesis were also demonstrated. Moreover, the results of the in vivo and in vitro tests show that the accelerated angiogenesis induced by OLAF is associated with the PI3K/Akt pathway.

Recently, oleosomes, as pharmaceutical formulations and good carriers of biological macromolecules, have attracted extensive attention [35]. Oleosomes are universal cellular organelles in plants composed of a hydrophobic neutral lipid core (triacylglycerols) surrounded by a monolayer phospholipid embedded with certain proteins [36]. Oleosins are the most abundant oleosome-associated protein in seeds, which include the central hydrophobic domain and the hydrophilic C-terminal and N-terminal domains [37]. In the central hydrophobic domain, a hairpin structure with a proline knot causes the oleosins to turn 180 degrees, which leads to the C-terminal and N-terminal domains being located on the surface of the oleosomes [38]. The physiochemical properties of oleosins and their association with oleosomes have led to the fusion expression of exogenous proteins with the C- or N-terminus [39]. Moreover, oleosomes can easily be purified by centrifugation, thus reducing the purification costs of exogenous proteins. Hence, multiple studies have attempted to express the various active proteins in plants using oleosin fusion technology, which offers distinct advantages in terms of the expression systems of bacteria, fungi, insects, etc. [40]. The successful expression of oleosin-GUS and oleosin-hirudin provide direct evidence for this deduction [24]. In our previous study, several medicinal proteins were successfully expressed in plant seeds using this technology, and the biological activity of some of the fusion proteins was proved. These results all prove the feasibility of this technology. However, there are no reports on the bioactivities of oleosomes fused with human fibroblast growth factor 1 (OLAF), especially their effect on wound vascular remodeling.

Here, full-thickness skin defect rat models were used to investigate the potential activities of OLAF, together with the underlying mechanisms of action. The results of this study show that OLAF is capable of accelerating wound healing. As is well-known, skin wound healing is an extremely complex process, which is divided into the inflammatory stage, proliferation stage and remodeling stage. Meanwhile, recent studies have found that nutritional items, including vitamins, amino acids and minerals, play a positive role in the whole process of wound healing [33]. It is reported that vitamin C and E have potential effects in promoting corneal wound healing [41]. Hu et al. prepared novel biomaterials loaded with vitamin E and proved their effectiveness in the healing of full-thickness skin defects [42]. Interestingly, there are also certain amounts of vitamin E and amino acids in oleosomes [43]. It is speculated that these components are conducive to wound healing, which may be related to the fact that the wound healing rate in the WTOB group was slightly higher than that in the model group. Moreover, oleosomes without hFGF1 were also found to accelerate the migration of HUVECs cells in vitro. Additionally, as an important skin lipid, triacylglycerol plays a prominent role in cell growth, differentiation, energy metabolism, etc. [44]. The cores of the oleosomes are also rich in triacylglycerol, which may also have a positive effect on the skin repair potential of the oleosomes without hFGF1. However, this weak effect of the oleosomes may be related to their low content of these components. In addition, the wounds treated with OLAF showed a weakened infiltration of the inflammatory cells, enhanced collagenation and angiogenesis, which further provide strong evidence for the wound healing efficiency of OLAF. Similarly, the oleosomes fused with other growth factors obtained by our group played an important role in collagen deposition and inflammatory cell infiltration in the wounds. However, little attention has been paid to the influence of recombinant oleosomes on angiogenesis during wound healing, especially the mechanism of this function, which has yet to be reported.

Emerging evidence suggests that uncontrolled vessel growth or impaired vessel regression can impair wound healing [45]. Thus, angiogenesis, the provider of nutrients and oxygen delivery, is considered a key component of wound healing. The current study attempted to explore the role of OLAF in angiogenesis in vivo and in vitro. In model rats, the effect of OLAF in accelerating angiogenesis was first demonstrated. Additionally, this role of OLAF was further confirmed by the CAM assay for the first time. CAM is formed by the fusion of the allantoic and chorionic mesoderm in avian species. Due to its rich vascular network for gas and waste exchange, CAM is often used to effectively study physiological and pathological angiogenesis. In addition, this in vivo model has also received extensive attention in the bioengineering and medicine fields, especially in the research on anti-tumor therapies, drug delivery, etc. The transparent appearance of CAM also makes it easier to observe the blood vessels within it [46]. Various test substances were proved to accelerate or inhibit angiogenesis in the CAM test platform [47,48,49]. For instance, the studies reported by Bai et al. demonstrated that the composite scaffolds containing growth factors could promote chorioallantoic angiogenesis in vivo [50]. In the present study, an obvious promotion of angiogenesis was observed in the CAM treated with OLAF, as well as a larger number of branches formed by endothelial cells. These data further confirm the positive correlations between OLAF and angiogenesis.

Undoubtedly, the process of angiogenesis is regulated at multiple levels, including the activation of intracellular signaling events [51]. In this process, the function of the vascular endothelial cells provides a substantial contribution to angiogenesis at the wound sites. Thus, the proliferation, migration, invasion and tube formation ability of OLAF-treated HUVECs were investigated here. Meanwhile, RNA-seq was introduced to analyze the HUVECs treated with the test substances, and a large number of DEGs were enriched in the PI3K/Akt signaling, which is an important pathway involved in the regulation of cell proliferation, migration, etc. [52]. The results of the Western blot detection revealed that, indeed, OLAF upregulated the protein expression of PI3K and p-Akt, which are similar to the effects of FGF1. It is well-known that FGF1 is a direct activator of PI3K and Akt and further stimulates the migration, invasion and differentiation of endothelial cells [53]. Forough and coworkers found that PI3K/Akt signaling was an important pathway involved in FGF1-stimulated angiogenesis [48,54]. Furthermore, the positive effect of OLAF on HUVEC proliferation was weakened in the cells pre-incubated with LY294002. These results reveal that the activation of PI3K/Akt signaling is required for OLAF-induced angiogenesis. Meanwhile, the expression of VEGF, a crucial angiogenic stimulator, also exhibited an induced enhancement by the OLAF treatment, and this effect was also weakened once the PI3K/Akt signaling was inhibited by LY294002. These findings indicate that the potential of OLAF to regulate angiogenesis is related to its activation of PI3K/Akt/VEGF signaling, which is consistent with other studies. Others’ researches also show the correlation between the expression of VEGF and the regulation of PI3K/Akt signaling. Human recombinant leptin was proved to promote the synthesis of VEGF via PI3K/Akt signaling in the CAM model [55]. Wang et al. demonstrated that integrin alpha x was involved in the regulation of VEGFR2/VEGF through PI3K/Akt signaling [56]. Additionally, Di et al. found that Rap2B was capable of promoting angiogenesis via the PI3K/Akt/VEGF signaling pathway in human renal cell carcinoma [57]. However, the underlying mechanism of OLAF that triggers PI3K/Akt signaling, regulating VEGF expression, remains to be explored in subsequent experiments.

## 4. Methods and Materials 

### 4.1. Oleosome Extraction Procedure

The seeds of wild *Arabidopsis thaliana* and transgenic *A. thaliana* expressing the human *FGF1* gene (GenBank accession No. BC03 2697.1) were both kept in our laboratory [30]. Gradient centrifugation was introduced to extract the oleosomes fused with or without hFGF1 from the seeds of transgenic or wild *A*. *thaliana* [31]. Briefly, the seeds were put into PBS solution (0.01 M) at a mass to volume ratio of 1 g to 385 mL and thoroughly ground. Following centrifugation for 20 min at 12,000× *g* (4 °C), the liquids in the upper two layers were separated and mixed together. Then, the above process was repeated three times to completely remove the precipitate. The oleosomes fused with human fibroblast growth factor 1 (OLAF) was harvested after centrifugation (4 °C, 12,000× *g* for 20 min).

### 4.2. Wound Healing Potential of Oleosomes Fused with hFGF1 in Full-Thickness Skin Defect Rats

#### 4.2.1. Creation and Treatment of Full-Thickness Skin Defect Rat Models

Healthy male Wistar rats (210 ± 10 g) were obtained from Changchun Yisi Experimental Animal Technology Co., Ltd. (SCXK (Ji)-2018-0007, Changchun, China) and used to establish the full-thickness skin defect rat models. In short, the dorsal hair of the rats was removed after anesthetization with sodium pentobarbital (40 mg/kg body weight). After disinfection, the skin was used to prepare two round full-thickness skin defects (1.77 cm^2^) using sterile surgical scissors, which were symmetrically distributed on both sides of the midline. Then, all the rats with full-thickness skin defects were fed in a single cage and randomly divided into four groups (n = 6). The wounds of the model rats were treated with 50 μL/cm^2^/d physiological saline (CONT group), 50 μL/cm^2^/d oleosomes (WTOB group), 1 μg/cm^2^/d commercial recombinant human acidic fibroblast growth factor for external use (POAF group, Tenry Pharmaceutical Co., Ltd, Shanghai, China) or 1 μg/cm^2^/d OLAF (OLAF group) for 15 days, respectively. The animal experiments were carried out under the guidance of the Institutional Animal Ethics Committee of Jilin Agricultural University (application approval number: 20200103006).

#### 4.2.2. Monitoring of Wound Closure and Specimen Collection

Digital photos of the wounds were obtained on days 0, 3, 6, 9, 12 and 15 after treatment with the different substances. Image J software (National Institutes of Health, Bethesda, MD, USA) was used to analyze the area of the wounds and was further used to analyze the healing rate of the wounds, as described in previous studies [58].

After applying the required substances for a different number of days, the experimental rats were anaesthetized by intraperitoneal injection of 1% pentobarbital, and the wounds with the surrounding skin were quickly harvested for further histological evaluation and immunohistochemical and immunofluorescence staining.

#### 4.2.3. Histopathological, Immunohistochemical and Immunofluorescence Assessment

To assess the pathological alterations in the wounds, hematoxylin and eosin (H&E) staining was carried out routinely, as previously described [59]. Additionally, Masson’s trichrome staining was conducted based on a previously described methodology to further assess the amount of collagen deposition in the wounds [60,61]. The stained sections were visualized and photographed using a microscope (Olympus IX51, Tokyo, Japan) and a digital camera (Canon Eos M6, Tokyo, Janpan), respectively. Images of the stained samples were analyzed via Image J software to assess the pathological changes in the wounds, including the wound bed width, epidermis thickness and the area of total collagen.

For the immunohistochemical and immunofluorescence analyses, after a series of treatments, including the deparaffinization, rehydration and retrieval of antigen blocked with 5% serum, the sections were, respectively, incubated with anti-CD68 antibody (Bioss, bs-1432R, 1:1000, Beijing, China), anti-CD31 antibody (Bioss, bs-0086R, 1:1000, Beijing, China) and anti-α-smooth muscle actin (α-SMA, Bioss, 1:1000, China) for 12 h at 4 °C. Subsequently, the sections were further incubated with secondary antibodies after washing with PBS. For the immunohistochemical analysis, goat anti-rabbit IgG/HRP antibody (Bioss, 1:1000, China) was used to further incubate the sections at room temperature. After one hour, 3,3-diaminobenzidine tetrahydrochloride (Solarbio, Beijing, China) and neutral resin were used to stain and fix the sections in turn. For the immunofluorescence analysis, a secondary antibody with goat anti-rabbit IgG/HRP (Bioss, 1:1000, China) was selected to incubate the sections. For the counterstaining, in order to visualize the cell nuclei, the sections were stained with DAPI solution (5 mg/mL). Finally, immunohistochemical images and immunofluorescence images were captured using a microscope (Olympus IX51, Japan) and a fluorescence microscope (NIKON ECLIPSE TI-SR, Tokyo, Japan), respectively.

Five microscopic fields per stained section for each of the abovementioned tests were randomly selected for the analysis of the CD68+, CD31+ and α-SMA+ cells using Image-Pro Plus (6.0) software (Media Cybernetics, Inc., Silver Springs, MD, USA). Then, the quantitation of the number of α-SMA+ pericytes, micro-vessel density (MVD) counts and the micro-vessel pericyte coverage index (MPI) of the wounds was further conducted [62].

#### 4.2.4. Evan’s Blue Assay

Evan’s blue assay, a common method with a high sensitivity and accuracy in analyzing the vascular permeability of animal tissues, was employed in this study [63]. Briefly, the rats with full-thickness skin defects were treated with 50 μL/cm^2^/d physiological saline (normal group) or 1 μg/cm^2^/d OLAF (OLAF group), according to the abovementioned steps, for 15 days and anesthetized at 12 h after the last administration. Then, Evan’s blue (5 mg/kg body weight) was injected into the tail vein of the rats. Forty minutes later, the wound areas were weighed and homogenated, and they were immediately put into 1 mL formamide and left at 37 °C for 48 h. Subsequently, the specimens were processed using centrifugation (3000 r/min, 15 min), and the supernatant was separated to detect its absorbance at 610 nm using a microplate reader (Tecan, Spark, Switzerland). The concentration of Evan’s blue in the specimens was calculated.

### 4.3. Chick Embryo Chorioallantoic Membrane (CAM) Assay for the Pro-Angiogenic Potential of OLAF

To further confirm the pro-angiogenic potential of OLAF, the CAM assay was introduced as a modified method used by Cao et al. [64]. In brief, fertilized chicken eggs (Liaocheng Longji Poultry Co., Ltd, Liaocheng, China) were incubated at 38 °C and in 65% humidity. Then, a 2 cm^2^ window was created above the air chamber of the eggs. After the removal of the shell membrane, 50 μL of different substances was added to the CAM of eggs aged 7 days, including physiological saline (CONT), oleosome without hFGF1 (WTOB), recombinant human acidic fibroblast growth factor for external use (3 μg, POAF) and 1, 2 and 3 μg oleosome with hFGF1 (OLAF). Untreated eggs were used as the normal control (NG group). Thereafter, the eggs, having been sealed with sterilized tape, were incubated for 3 days under the abovementioned conditions. A minimum of 7 eggs in each group were used. The CAM was photographed using a digital camera, and the analyses of the vessels area (VA), vessel density (VD), total number of junctions (TNJ) and total vessel length (TVL) were performed using the AngioTool software (National Cancer Institute, Bethesda, Maryland). Then, the CAM was separated for the H&E staining to analyze the angiogenesis.

### 4.4. In Vitro Evaluation of the Beneficial Effect of OLAF on Angiogenesis

#### 4.4.1. Cell Culture

Human umbilical vein endothelial cells (HUVECs), supplied by Procell Life Science & Technology Co., Ltd., (Wuhan, China), were maintained in an incubator with 5% CO_2_ at 37 °C in Dulbecco’s modified Eagle’s medium (Solarbio, Beijing, China) with fetal bovine serum (FBS) and penicillin–streptomycin in proportions of 10% and 1%, respectively [65].

#### 4.4.2. Cell Proliferation Vitality Assessment and Morphological Observation

For the proliferation vitality assay, HUVECs were added to 96-well plates (5 × 10^3^ cells/well) and maintained as described above. Twenty-four hours later, the cells were incubated with/without LY294002 (phosphatidylinositol 3-kinase inhibitor, 10 μmol/L) for one hour in advance of the treatments, using different test substances for an additional twenty-four hours. The four test substances were physiological saline (100 μL, CONT), oleosome without hFGF1 (100 μL, WTOB), recombinant human acidic fibroblast growth factor (100 μL, 100 ng/mL POCT) and oleosome with hFGF1 (100 μL, 100 ng/mL OLAF). Subsequently, the viability of the HUVECs was detected using the commercially available Cell Counting Kit-8 (CCK-8), as per the recommendations of the manufacturer (Solarbio, Beijing, China). After incubation with the CCK-8 solution for two hours, the absorbance at 450 nm was detected using a microplate reader (Tecan, Spark, Switzerland). Meanwhile, the morphology of the acridine-orange (AO)-stained or unstained cells was observed using a fluorescence microscope (Olympus IX51, Tokyo, Japan).

Additionally, Ki-67 immunofluorescence staining was introduced, according to previous studies, to assess the function of OLAF in accelerating the proliferation of the HUVECs [66]. Briefly, after fixation, permeabilization and Ki-67 antibody (Bioss, bsm-33070M, 1 mg/mL) incubation, the cells treated with various substances were further washed with PBS, and this procedure was repeated three times. Then, the cells were allowed to incubate with the secondary antibody conjugated with Alexa-Fluor-488-conjugated anti-mouse IgG secondary antibody (Abcam, 150113, 1:200, Cambridge, UK). Next, mounting buffer containing DAPI was used to embed the slices. Each of the above experiments were conducted in 3 replicates.

#### 4.4.3. Scratch Wound Assay and Transwell Migration Test

The scratch wound assay was carried out as per a modified method described previously [67]. Cells grown on a plate (6 wells, 1 × 10^5^/well) were allowed to grow in an incubator for 24 h. Monolayer cells were mechanically scratched using a sterile yellow pipette tip and then continued to be cultured in serum-free medium containing POCT (100 ng/mL) or OLAF (100 ng/mL) together with mitomycin C (5 µg/mL). The cells treated with the test substances for 0 and 24 h were photographed, and the migration rate of the cells was calculated.

The transwell migration assay is a system that is widely used to study the cell migration ability [68]. For the transwell migration assay, the transwell chambers contained polycarbonate filters (Labselect, Lot:14341, Hefei, China) that were employed in the experiments. The HUVECs (1.5 × 10^4^ in 200 μL serum-free DMEM medium containing 5 µg/mL of mitomycin C) were added to the upper chamber. Subsequently, medium (600 µL, 20% FBS) containing OLAF (100 ng/mL), POCT (100 ng/mL) or a vehicle was added to the bottom chamber. Then, the transwell chambers were put in an incubator, and 24 or 48 h later, the upper chamber was taken out, and the medium was removed. After this step, the cells in the upper chamber were treated using the following process: removal with cotton swabs, fixation with 4% paraformaldehyde (20 min), crystal violet staining (20 min) and repeated washing with PBS three times. After drying, the observation of the cells was conducted using a microscope, and the numbers of cell-permeating septa in the five fields were counted.

#### 4.4.4. Tube Formation Assay

The tube formation assay was introduced to investigate the angiogenic potential of the HUVECs [69]. Briefly, 0.1 mL HUVECs with a density of 3 × 10^4^/mL were seeded onto 48-well plates that were pre-coated with Matrigel (BD Bioscience, CA, USA). After six hours of culture with the test substances, as mentioned in Section 4.4.2, images were taken of each well using a microscope (Olympus IX51, Tokyo, Japan). Image Pro Plus 6.0 was used to analyze the average number of capillary-like branches, tube length, etc.

### 4.5. RNA-Seq-Based Transcriptome Analysis of HUVECs under OLAF Treatment

In order to evaluate the changes in the gene expression profiles of the HUVECs treated with OLAF, the cells exposed with/without 100 ng/mL OLAF for 1 day were sent to Novogene Bioinformatics Technology Co., Ltd., (Beijing, China) for transcriptome sequencing and analysis (three biological replicates per treatment group). In brief, the total RNA of the cells was extracted using TRIzol reagent, and its purity and integrity were further detected using a NanoDrop Spectrophotometer (Peqlab ND-1000, CA, USA) and an Agilent 2100 Bioanalyzer (Agilent Technologies, Santa Clara, CA, USA), respectively. Then, purified mRNA was used to construct the cDNA libraries, which were further sequenced by 150 bp paired-ended sequencing on an Illumina Novaseq platform. Subsequently, after obtaining the clean reads, the fragments per kilobase of exon per million fragments mapped (FPKM) were employed to normalize the read counts. Then, the differentially expressed genes (DEGs) were identified using the DESeq2 package (1.20.0), with a threshold *p*-value of less than 0.05. For the functional annotation and metabolic pathway analysis of the DEGs, the Gene Ontology (GO) database and Kyoto Encyclopedia of Genes and Genomes (KEGG) databases were used.

### 4.6. Western Blot and qRT-PCR

RNAiso plus (Takara, Dalian, China) was employed to separate the total protein from the wounds or HUVECs. After separation by polyacrylamide gel electrophoresis, 100 mg of protein was transferred to polyvinylidene fluoride membranes, which were subsequently blocked with 5% nonfat milk for 1 h. Afterwards, primary antibodies against PI3K, AKT, p-AKT, VEGF, VEGFR2, ANG-1, Tie-2 and hFGF1 (Bioss, 1:1000, Beijing, China) were introduced for incubation with the PVDF membranes overnight at 4 °C. Then, horseradish-peroxidase-labeled antibody (Bioss, 1:1000, Beijing, China) was used as the secondary antibody to incubate the membranes for 1 h at 25 °C. For the internal control, β-actin was employed. The intensities of the protein bands were analyzed by the Universal hood II imaging system (Bio-Rad, Hercules, CA, USA).

A qRT-PCR analysis was carried out on a Stratagene Mx3000P thermocycler (Agilent Technologies, Santa Clara, CA, USA) using a commercially available kit (SYBR Premix Ex TaqTM II Kit, Takara, China), and the relative expression levels of the genes in the samples were analyzed using a comparative threshold (2^−ΔΔCt^). The primers of the genes used in this test are shown in Table S1.

### 4.7. Statistical Analysis

The data are represented as the mean ± standard deviation (SD). GraphPad Prism 8.0 software (GraphPad Software, San Diego, CA, USA) was used to perform one-way ANOVA analyses followed by post hoc multiple comparisons (Dunn’s test), and significant statistical differences were identified when a *p*-value less than 0.05 was found.

## 5. Conclusions

Taken together, oleosomes fused with hFGF1 (OLAF), a new transdermal delivery substance, proved to be potential preparations for promoting the healing of skin wounds by alleviating inflammation and accelerating collagen deposition at the wound site. More importantly, the role of OLAF in improving wound vascular network reconstruction by accelerating angiogenesis was also clarified in vivo and in vitro, which might be explained by the upregulation of the expression of VEGF through the activation of the PI3K/Akt signaling axis. Hence, the OLAF developed in our previous study shows great potential for application as a candidate drug for the treatment of skin injury.

## Supplementary Materials

The following supporting information can be downloaded at: https://www.mdpi.com/article/10.3390/ijms232113152/s1.
