# Supplementary material for: Beneficial Effects of Oleosomes Fused with Human Fibroblast Growth Factor 1 on Wound Healing via the Promotion of Angiogenesis
Source: Int J Mol Sci. 2022 Oct 29;23(21):13152. doi: 10.3390/ijms232113152 (PMC9656666; doi:10.3390/ijms232113152)
Supplement: Supplementary file 1 [file ijms-23-13152-s001.zip › ijms-1932425-supplementary.pdf]

International Journal of *Molecular Sciences*

MDPI

Supplementary Materials

Table S1. Gene specific primers used in qRT–PCR

Gene	Forward Primer (5′-3′)	Reverse Primer (3′-5′)
β-Actin	AGAGCTACGAGCTGCCTGAC	AGCACTGTGTTGGCGTACAG
Ang-1	AAAGGTCACACTGGGACAGC	TTCTGACATTGCGCTTTCAA
Tie-2	CTGCGGCATGACATGTGCAG	GCAAATGATGGTCTCTCATAAGG
VEGF	TACCTCCACCATGCCAAG	GGTACTCCTGGAAGATGTC

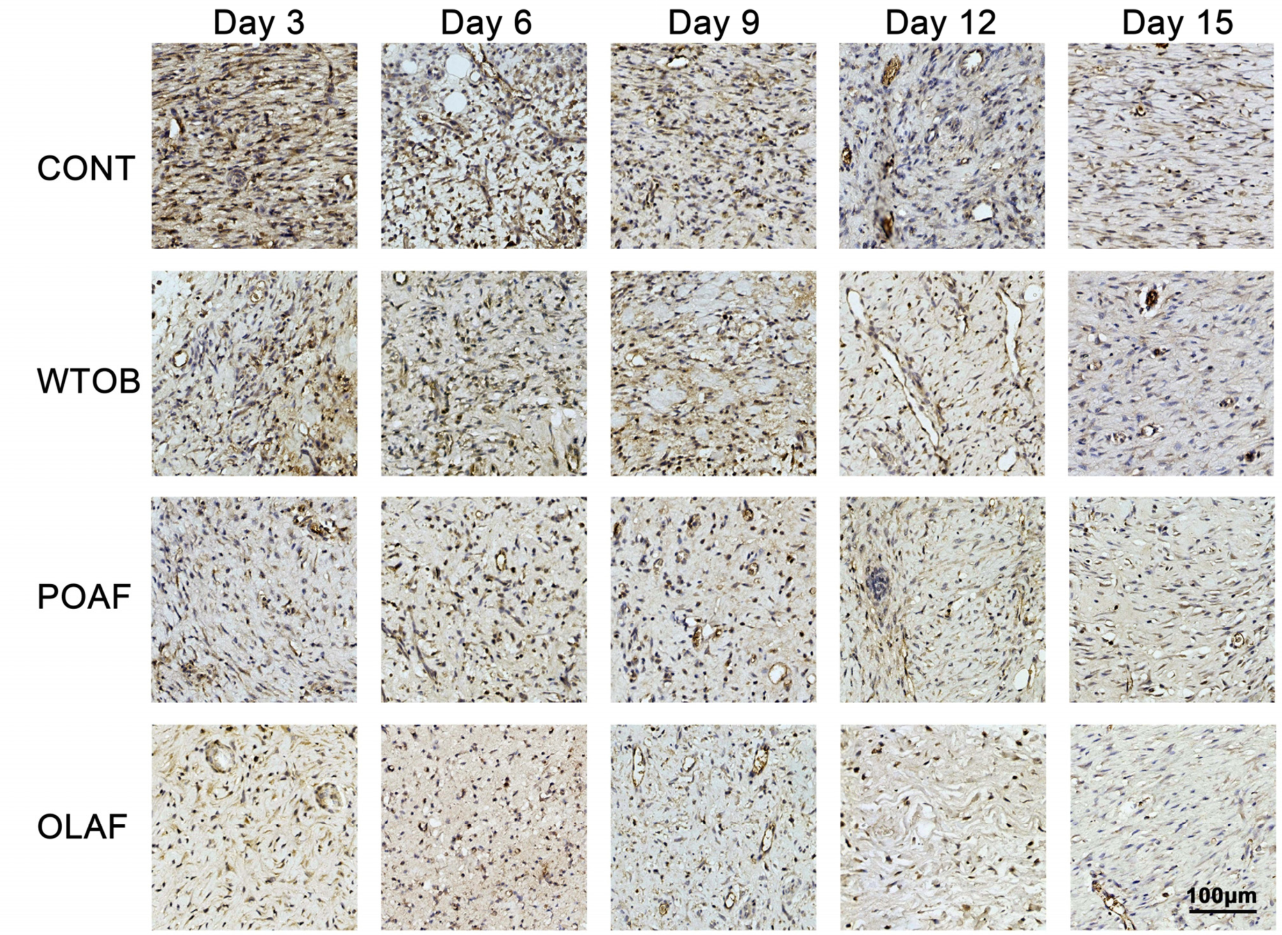

Figure S1. Representative images of immunohistochemical staining for CD68 positive cells in wounds. CD68+ cells were stained brown.

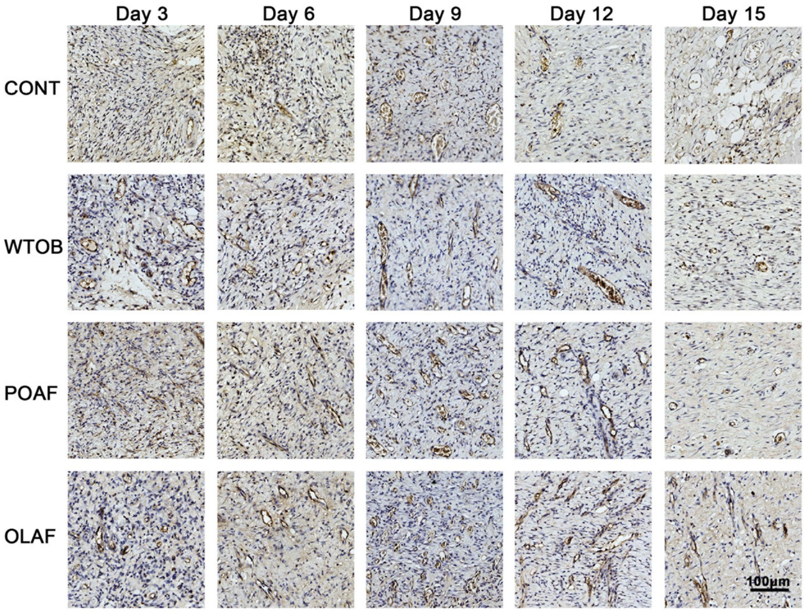

Figure S2. Representative images of immunohistochemical staining for CD31 positive cells in wounds. CD31+ cells were stained brown.

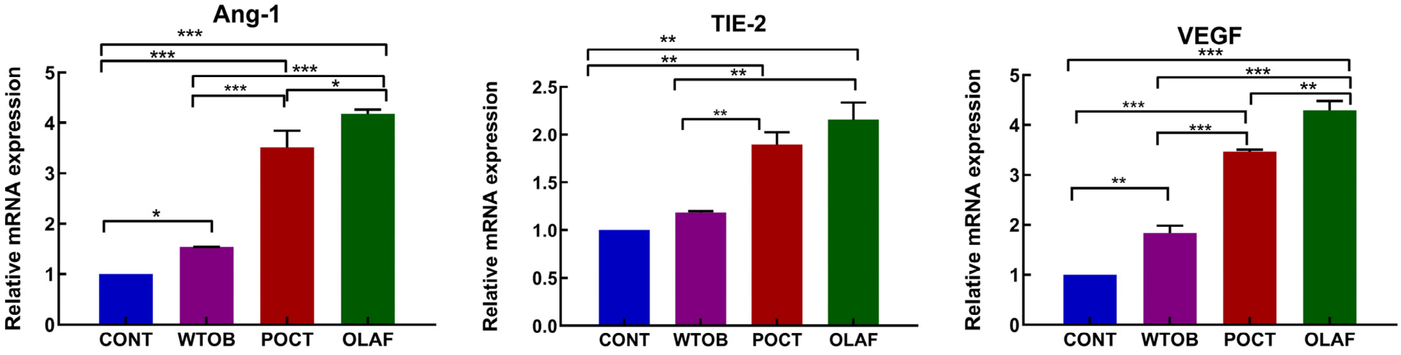

Figure S3. The relative expression levels of selected genes were determined in HUVECs treated with physiological saline (CONT), oleosome without hFGF1 (WTOB), recombinant human acidic fibroblast growth factor (POCT) and oleosome with hFGF1 (OLAF) for 24 hour, respectively. * $p < 0.05$, ** $p < 0.01$, *** $p < 0.001$. Means ± SD was employed to display all obtained data (n = 6).
